# Hypertensive emergency and seizures in a 30‐year‐old man with anti–glomerular basement membrane disease

**DOI:** 10.1002/ccr3.3361

**Published:** 2020-10-20

**Authors:** Michael Mascola, Rehan Karmali, Joshua Mathews, Sergio Obligado

**Affiliations:** ^1^ Touro College of Osteopathic Medicine Middletown NY USA; ^2^ Garnet Health Medical Center Middletown NY USA

**Keywords:** anti–glomerular basement membrane disease, hypertensive emergency, seizure

## Abstract

Anti–glomerular basement membrane disease can rapidly lead to renal failure and blood pressure dysregulation. A rare complication is hypertensive encephalopathy in the form of seizures. Patients who have a negative initial seizure workup should have an MRI. These patients need tight blood pressure control and monitoring to prevent future seizures.

## Graphical abstract

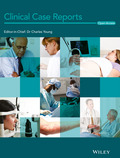

## 1 INTRODUCTION

Anti–glomerular basement membrane (anti‐GBM) disease is a form of vasculitis where autoantibodies attack antigens of the glomerular basement membrane leading to rapidly progressive glomerulonephritis.^1^ The complications surrounding this disease often circulate around renal and pulmonary pathologies. There is some mention of neurological complications associated with anti‐GBM disease in literature, but the actual pathologies and mechanisms remain unclear. Some of the possible causes include cerebral vasculitis, metabolic encephalopathy, or posterior reversible encephalopathy syndrome (PRES).^2^ The pathogenesis of neurological symptoms in a patient with anti‐GBM disease such as hypertensive emergency in the form of hypertensive encephalopathy is clinically significant in patients with severe renal disease. Hypertensive encephalopathy is a diagnosis of exclusion and thus involves extensive workup to rule out all other etiologies of neurologic dysfunction and BP dysregulation.^3^ Acute or chronic kidney disease, including anti–glomerular basement membrane disease (anti‐GBM), is among well‐known causes of hypertension and can lead to hypertensive emergencies.^4^, ^5^ Here, we present a patient newly diagnosed with anti‐GBM disease complicated by end‐stage renal disease (ESRD) who had two hospital admissions for hypertensive emergency with seizures. Our workup, investigations, and treatment suggest hypertensive encephalopathy as the cause of seizures in our patient.

## 2 CASE PRESENTATION

This is a 30‐year‐old Caucasian man with biopsy‐confirmed glomerular basement disease diagnosed 3 months prior to presentation, with past medical history of tobacco use disorder. He received treatment with hemodialysis (HD), glucocorticoids, and oral cyclophosphamide for anti‐GBM disease treatment, as well as valsartan, amlodipine, and carvedilol for blood pressure control. He presented to the ED for nausea and nonbloody nonbilious vomiting with associated frontal headaches and photophobia. His BP was 184/124 and physical examination showed right lower quadrant tenderness, but otherwise was unremarkable and without any focal neurological deficits. His course was complicated by two witnessed grand mal seizures in the ED lasting 45 seconds and 25 seconds, respectively, which were controlled with lorazepam and levetiracetam. He was admitted to the intensive care unit (ICU) where his BP was controlled by the addition of clonidine and bumetanide to his outpatient regimen. Amlodipine was discontinued on admission due to concern that it may have caused immune thrombocytopenic purpura (ITP) as he was thrombocytopenic (Table 1). He was eventually stabilized and kept in the hospital for 3 days for further workup. He was ultimately discharged on valsartan, carvedilol, clonidine, minoxidil, bumetanide, and continued HD.

**TABLE 1:** Laboratory results from hospital admissions requiring seizure workup

Test	Reference range	First seizure admission laboratory value	Second seizure admission laboratory value
Red blood cells	4.3‐5.8 × 10^6^/uL	2.93	4.19
Hemoglobin	13.3‐17 g/dL	9.1	12.3
Hematocrit	40.3%‐50.3%	27.9	37.7
Red cell distribution width	11.6%‐14.9%	17.6	12.6
Platelets	132‐337 × 10^3^/uL	58	324
White blood cells	4.3‐10.6 × 10^3^/uL	10.6	13.0
Mean corpuscular volume	81.4‐99.0 fL	95.2	90.0
Sodium	136‐144 mEq/L	140	137
Potassium	3.5‐5.1 mEq/L	4.2	4.2
Chloride	101‐111 mEq/L	99	96
Carbon dioxide	22‐32 mEq/L	24	14
Blood urea nitrogen	8‐20 mg/dL	49	61
Glucose	70‐110 mg/dL	116	135
Calcium	8.5‐10.1 mg/dL	8.5	9.7
Creatinine	0.55‐1.02 mg/dL	7.50	13.43
Anion gap	8‐17 mEq/L	17	27
Glomerular filtration rate	>60.0 mL/min/1.732 m^2^	8.6	‐
Albumin	3.5‐4.8 g/dL	2.9	‐
total protein	6.4‐8.3 g/dL	4.8	‐
Total bilirubin	0.3‐1.2 mg/dL	0.8	‐
Direct bilirubin	0.10‐0.50 mg/dL	<0.10	‐
Alkaline phosphatase	45‐117 U/L	45	‐
Aspartate aminotransferase	15‐41 U/L	21	‐
Alanine aminotransferase	17‐63 U/L	11	‐
Lactic acid	0.5‐2.2 mmol/L	1.2	>10.0
Troponin	<0.03 ng/mL	0.06	0.07
Reticulocytes	0.60%‐2.40%	7.03	‐
Haptoglobin	43‐212 mg/dL	11	‐
Vitamin B12	180‐914 pg/mL	925	‐
Folate	>6.6 ng/mL	> 20.0	‐
Ferritin	24‐336 ng/mL	233	‐
Fibrinogen	200‐393 mg/dL	238	‐
Lactate dehydrogenase	87‐241 u/L	359	‐

Two months later, the patient was brought in by emergency medicine services (EMS) after an unwitnessed fall and his second presumed seizure. At this time, he was still on HD three times per week (prednisone and cyclophosphamide had been discontinued) and his BP regimen was adjusted in the outpatient setting to losartan, carvedilol, clonidine, and minoxidil. His family heard a loud thud from another room and found him on the floor minimally responsive but awake. On EMS arrival, the patient was complaining of a headache and was noted to be mentally altered. His BP at that time was 218/130 and on route to the ED, EMS witnessed a grand mal seizure of 1‐minute duration. In the ED, his BP remained elevated at 209/137 and he was mildly confused and agitated, presumed due to postictal state. He had a GCS of 15, 5/5 muscle strength in all extremities, intact sensation to light touch, and no focal neurological deficits. He received multiple doses of lorazepam in the ED to combat seizure activity and was intubated for airway protection. He was then admitted to the ICU for further seizure workup and blood pressure control with a nitroglycerin drip.

## 3 INVESTIGATIONS AND DIFFERENTIAL DIAGNOSIS

Initial differential diagnosis for the patient's new‐onset seizures included hemorrhagic stroke, ischemic stroke, new‐onset epilepsy, metabolic encephalopathy, hypertensive encephalopathy, hepatic encephalopathy, hypertensive emergency, acute drug/alcohol intoxication, and PRES. The medical workup of both seizure admissions was combined in the following sections for simplicity.

### 3.1 Neurology

Computerized tomography (CT) of the brain without contrast on both admissions was negative for acute intracranial pathology. The ventricles and sulci were normal and symmetric bilaterally and there was no evidence of hemorrhage, infarction, or mass ruling out stroke or tumor. There were no signs of cerebral edema ruling out PRES. VEEG monitoring was performed for several continuous days during both admissions and was inconclusive. There were no asymmetries, no seizures, no epileptiform abnormalities, and no periodic patterns. There was mixed frequency slowing but the neurology team suspected this to be from sedation as the patient remained intubated and on mechanical ventilation. Neurology ultimately found no clear etiology for his seizures ruling out epilepsy. MRI was not obtained during either of his admissions.

### 3.2 Nephrology

Blood ethanol and urine toxicology were unremarkable. He continued to receive HD on a three times per week schedule. Repeat basic metabolic panel continued to show impaired renal function (Table 1). Mild fluid overload (possibly incorrect estimated dry weight) due to his severe kidney disease likely contributed to his uncontrolled BP at home.

### 3.3 Hematology

During the second admission, when the patient first presented with seizures there was suspicion for thrombotic microangiopathy possibly due to severe hypertension, ITP, or thrombotic thrombocytopenic purpura (TTP). Platelet count was 58 000 and lactate dehydrogenase was 359 but trended down. He had a negative thrombotic workup (Table 1) consisting of normal ADAMTS13 activity, negative direct antiglobulin test, normal serum C4, normal serum C3, parasite smear, and iron studies. Laboratory results were notable for a high reticulocyte count and low haptoglobin suggestive of hemolysis. The hepatitis panel was negative on multiple occasions. Prior to discharge, his platelet level stabilized and he was asymptomatic where we ultimately came to a diagnosis of thrombotic microangiopathy secondary to HTN.

### 3.4 Infectious disease

Infectious disease was consulted as he had leukocytosis with a white count of 13 000 and lactic acidosis but no source of infection. Repeat CBCs showed that leukocytosis had resolved on its own. Elevated lactic acid resolved and was likely elevated due to seizure activity as opposed to infectious process. Blood cultures showed no growth through admission. One urinalysis showed some bacteria but also squamous cells; thus, we could not diagnose him with a UTI definitively. Lumbar puncture with CSF was considered but not done as there were no neurological deficits and the patient improved once BP was controlled. Ultimately, we did not suspect an infectious diagnosis to be contributing to this patient's case.

### 3.5 Cardiology

EKGs were obtained; one of them showed occasional PVCs and at one point he had a slightly prolonged QT at 503 ms but otherwise unremarkable. Troponin was mildly elevated but eventually down trended with elevation likely due to impaired clearance of troponin due to ESR.

## 4 TREATMENT

Since an underlying cause of his seizures was not identified after thorough workup, treatment focused on controlling the patient's BP with medications and optimizing his volume status with dialysis continuing ESRD treatments. There was concern that he may have been missing BP medication doses at home resulting in large increases in BP and hence, was advised to continue losartan, carvedilol, clonidine, and minoxidil with the clonidine being in the form of a catapres patch. Given the unknown etiology of seizures, although most likely from hypertensive emergency and subsequent encephalopathy, the patient was discharged home on levetiracetam to help prevent future seizures.

## 5 DISCUSSION

There are many etiologies that have been known to cause seizures. These include epilepsy, hemorrhagic stroke, cerebral hypoxia, metabolic derangement, thyroid disease, head trauma, CNS infection, vascular malformations, intoxication, poisoning, and some of the rarer causes include brain tumors, hypertensive encephalopathy, or PRES.^6^

Our patient had an extensive seizure workup during his hospital admissions. One of the most important tests to perform is VEEG as this provides insight as to the type of seizure a patient may be having and if there is underlying epilepsy.^7^, ^8^ Continuous VEEG was performed on our patient during both admissions for seizures. Obtaining continuous VEEG monitoring or at least four separate VEEGs increases the chance of picking up underlying epileptic pathology by about 80%‐90%.^9^, ^10^ Continuous VEEG in our patient never identified any waveforms suggestive of epilepsy. Neuroimaging is another crucial component of the seizure workup. Typically, brain magnetic resonance imaging (MRI) is preferred over CT scan as MRI has higher sensitivity for epileptogenic lesions as well as having superior soft tissue contrast for identifying any other possible underlying pathology, but CT scans are often sufficient enough to make an accurate diagnosis.^7^, ^11^, ^12^ Our patient had CT scans of the brain on both presentations but showed no abnormality suggesting the cause of his seizures. In this patient, CT scans of the brain were preferred over MRI originally given the patient acuity as CT scans can be obtained quicker than an MRI and are often sufficient in identifying any potential causes of seizures.^7^, ^8^, ^13^ Typically, seizure workup can be continued with outpatient MRI; however, in our case it was deferred as CT brain and VEEG were normal and an MRI was not going to change our treatment plan especially since the patient did not have any additional seizures once his blood pressure was controlled.

Additional workup of seizures includes blood work to evaluate metabolic and thyroid issues and ECG and lumbar puncture to rule out hypoxic cerebral injury from syncope and infectious processes, respectively.^7^, ^14^, ^15^ Although there were some abnormalities in his laboratory results, there was nothing evident as the cause of his seizures (Table 1). ECGs were done on our patient and were insignificant. Moreover, since there were no infectious signs or symptoms and blood cultures never showed any growth of organisms, a lumbar puncture was not indicated.

Given the extensive negative neurologic, hematologic, and infectious workup, seizures in our patient likely occurred due to hypertensive encephalopathy from increased intracranial pressure. The cerebral perfusion pressure (CPP) of the brain has a narrow and sensitive window that is easily affected by changes in systemic BP. Normally when there is an increase in systemic BP, the cerebral vasculature vasoconstricts to maintain CPP in physiologic range. In sudden increases of systemic BP or uncontrolled hypertension, CPP is not maintained and can lead to cerebral edema resulting in neurologic dysfunction manifesting as seizures.^16^, ^17^ After our patient's blood pressure was stabilized during admission, he did not have any additional seizures suggesting that the cause of his seizures was severely elevated blood pressures.

Acute or chronic kidney disease has been shown to cause hypertensive emergency and encephalopathy.^4^, ^5^ 80%‐85% of patients with chronic kidney disease (CKD) have hypertension.^18^ Hypervolemia is due to decreased glomerular filtration rate (GFR), which results in sodium retention and extracellular fluid expansion.^19^ Furthermore, an overactive renin‐angiotensin system due to renal ischemia can lead to increased angiotensin II and renin causing increased systemic BP, while uremia and decreased renal clearance can cause a neural reflex that increase levels of the vasoconstrictor endothelin.^20^ Some of the implicated renal diseases that can follow this physiology include acute glomerulonephritis, renal vascular disease, renal infarction, and renal failure. Anti‐GBM disease is a rapidly progressive glomerulonephritis falling under this category.

End‐stage renal disease patients on HD have a high prevalence of hypertension and seizures.^21^, ^22^ HD‐related complications may partly underlie our patient's disease course, but do not provide a complete explanation for his recurrent admissions. Our patient was compliant with his medications and outpatient HD prior to both admissions and did not have any episodes of hypertensive encephalopathy during both hospital stays when receiving HD. Interestingly, the collagen antigens eliciting an autoimmune response in anti‐GBM disease are found not only in the alveolar and renal basement membranes, but also in the choroid plexus in the brain.^23^, ^24^ Hence, possible neural antigenic cross‐reactivity may also explain his encephalopathy. An additional possibility not primarily considered during his workup was PRES. PRES is a clinical syndrome consisting of headache, altered mental status, visual changes, and seizures resulting from cerebral edema and disrupted autoregulation of blood pressure.^25^ Our patient had multiple known risk factors for PRES including immunosuppressive therapy, hypertension, and glomerular disease.^25^, ^26^ CT findings in press include cerebral white matter edema usually in the posterior cerebral hemispheres, but our patient's CT was unremarkable. In retrospect, an MRI may have helped diagnose PRES as well as visualize any autoimmune vasculitic changes. However, it was not ordered for our patient as it would not change our treatment plan.

Regarding treatment, initial management of this patient was aimed at controlling the seizure. Patients experiencing seizures without a history of epilepsy are typically treated with a single antiepileptic medication.^27^ Our patient received lorazepam to terminate the seizure and levetiracetam to prevent recurrence of seizures. Typically, seizures due to underlying metabolic derangements or nonepileptic disease do not require maintenance therapy. Rather, these patients require adequate control of the underlying disease causing the seizures. But patients with severe underlying disease may benefit from a short course of antiepileptic medication to prevent recurrent seizures or further complications.^28^ Given our patient's return to the ED with poorly controlled BP and ongoing HD, he was at an increased risk of recurrent seizures and thus levetiracetam therapy for seizure prophylaxis was prescribed. Levetiracetam shown to be more effective with a safer side effect profile compared to other prophylactic seizure medications.^16^ The decision to continue prophylaxis following discharge was based on clinical efficacy, absence of adverse side effects, and tenuous blood pressure control on four antihypertensives in the setting of ESRD and ongoing hemodialysis. The patient is instructed to routinely follow‐up monthly to risk stratify for seizure recurrence and determine the need of continued prophylaxis.

Controlling BP in hypertensive emergency with hypertensive encephalopathy and ESRD varies by physician preference, although most of the treatments focus around IV antihypertensives for rapid control.^29^ Our patient's BP was effectively treated at the first seizure admission with clonidine and his PTA medications (losartan, carvedilol, and minoxidil). On the second seizure admission, he required a nitroglycerin drip which is commonly used in hypertensive emergency and was effective at controlling his BP.^29^ We suspect the underlying cause of our patient's hypertensive episodes to be the frequently resistant nature of hypertension in patients with ESRD in combination with anti‐GBM disease. Hence, he was also on levetiracetam prophylaxis and did not have any additional seizures after his BP was controlled. In addition, with BP medication compliance as an outpatient, there have been no further reports of additional seizures since his last admission.

Given the rarity of anti‐GBM disease and its unique complications, treatment in a rural setting can be challenging. Transfer to a facility with higher level care is necessary to acutely stabilize the patient. A combination of telemedicine to bridge access to care, in‐home dialysis, and community‐based rehabilitation can help prevent recurrence of life‐threatening complications like hypertensive emergency and seizures.^30^, ^31^, ^32^

## 6 CONCLUSION

Our patient was newly diagnosed with anti‐GBM disease and presented with the unique pathophysiology of hypertensive emergency and subsequent encephalopathy in the form of seizures. He was successfully stabilized and managed with strict blood pressure and seizure control. Our report brings attention to the complications that may arise with anti‐GBM disease. Our patient has not had any recurrence of seizures implying that the cause of his seizures was most likely related to his hypertensive crises and that adequate BP control can prevent seizures in patients with anti‐GBM disease.

## CONFLICTS OF INTEREST

The authors declare no conflicts of interest.

## AUTHOR CONTRIBUTIONS

MM: initiated this case report, performed patient chart review, performed literature review, and wrote first and final manuscript. RK: performed literature review and assisted in writing and editing the manuscript. SO: performed patient chart review and assisted in writing and editing this manuscript. JM: performed literature review and assisted in writing this manuscript.

## ETHICAL APPROVAL

This case report does not contain any clinical studies with human participants performed by any of the authors.

## CONSENT

Written consent for publication of this case report and accompanying images was obtained from the patient.
